# Chronic Steroid Use Does Not Increase the Risk of Superficial Surgical Site Infection or Wound Dehiscence Following Total Ankle Arthroplasty

**DOI:** 10.7759/cureus.52569

**Published:** 2024-01-19

**Authors:** Alexander R Garcia, Kenny Ling, Evan Olsen, David E Komatsu, Megan Paulus

**Affiliations:** 1 Orthopaedic Surgery, Stony Brook University, New York, USA

**Keywords:** sepsis, chronic steroid use, ankle arthrodesis, ankle arthritis, total ankle arthroplasty

## Abstract

Introduction

Total ankle arthroplasty (TAA) is an effective treatment for end-stage ankle arthritis. Recent surgical and technological advances have led to a significant increase in the surgical volume of TAA. While a majority of ankle arthritis is post-traumatic in nature, other causes include autoimmune or inflammatory conditions. Medical management of these conditions frequently requires chronic corticosteroid administration, which is a well-established risk factor for complications following surgery. The purpose of this study was to investigate the association between chronic preoperative steroid use and postoperative complications following TAA.

Methods

The American College of Surgeons National Surgical Quality Improvement (NSQIP) database was analyzed to identify all patients who underwent TAA between 2015 and 2020. Patient characteristics including demographics, comorbidities, surgical characteristics, and 30-day postoperative complication data were collected. The data was analyzed using bivariate and multivariate logistic regression to identify all postoperative complications associated with chronic preoperative steroid use.

Results

A total of 1,606 patients were included in this study: 1,533 (95.5%) were included in the non-steroid cohort, and 73 (4.5%) were included in the chronic steroid cohort. Chronic steroid use was significantly associated with female sex (p < 0.001) and American Society of Anesthesiologists (ASA) ≥3 (p < 0.001). Chronic steroid use was not associated with superficial surgical site infection (SSI) (p = 0.634) or wound dehiscence (p = 0.999). The postoperative complication that was significantly associated with chronic steroid use was sepsis (p = 0.031). After adjusting for female sex and the ASA grade, chronic steroid use was found to be independently associated with sepsis (p = 0.013).

Conclusion

Preoperative chronic steroid use is not associated with superficial SSI or wound dehiscence within 30 days following TAA. As TAA becomes a more attractive alternative to ankle arthrodesis, a better understanding of preoperative risk factors can aid in widening indications and knowing what patients are at risk for complications.

## Introduction

Total ankle arthroplasty (TAA) has emerged as an effective alternative to the historical “gold standard” of ankle arthrodesis (AA) for advanced ankle arthritis due to significant improvements in implants [1-3]. According to a Nationwide Inpatient Sample study, the use of TAA has more than doubled from 390 TAA procedures in 1998 to 844 being performed in 2010 [4]. Although TAA has demonstrated the major benefits of better functional recovery and decreased radiographic incidence of adjacent joint degeneration compared to AA, the reoperation rate remains higher than arthrodesis. Therefore, it is important that patients are evaluated on a case-by-case basis to decide between TAA and AA [1,3,5-7].

End-stage ankle arthritis is a major indication for TAA and AA. Unlike hip and knee arthritis, 70% of ankle arthritis is post-traumatic [8]. Compared to total hip and total knee arthroplasty, TAA has more frequent complications such as inadequate wound healing and malleolar fractures [9]. Other causes of ankle arthritis include mechanical wear and tear or inflammatory or autoimmune conditions [8]. Management of inflammatory conditions can include chronic corticosteroid administration, which has been shown to affect long-term survival rates in hip arthroplasty, in addition to being associated with delayed wound healing and increased rates of superficial and deep surgical site infections (SSIs) after surgery [10-15]. Although chronic steroid use may be less frequent in TAA patients due to the more traumatic nature of ankle arthritis, the inherent postoperative risk factors associated with TAA make it important to investigate whether chronic steroid use increases the risk of postoperative complications.

Previous studies have investigated the association of chronic steroid use and postoperative complications in lumbar fusion, knee, hip, and shoulder arthroplasty [10,12,16-18]. Chronic steroid use was found to be an independent risk factor for SSIs, pneumonia, urinary tract infections, and readmission for all of these procedures. However, there have been no investigations into the association between preoperative chronic steroid use and postoperative complications following TAA. As TAA has previously demonstrated a higher rate of reoperation than AA due to deep infection, this investigation can aid in the decision-making process of proceeding with TAA versus AA [19].

The purpose of this study was to investigate preoperative chronic steroid use and its association with postoperative complications following TAA to improve perioperative planning and risk stratification. We hypothesized that chronic steroid use would be associated with increased rates of postoperative infections following TAA.

## Materials and methods

This study identified patients from the American College of Surgeons National Surgical Quality Improvement Program (ACS-NSQIP) database who underwent TAA from 2015 to 2020. This study was exempt from approval by our university’s Institutional Review Board as it only utilized fully deidentified info from the NSQIP database. Over 600 hospitals in the United States contribute to the NSQIP database, and data was obtained by trained surgical clinical reviewers.

Current Procedural Terminology (CPT) codes 27700, 27702, and 27703 were used to identify patients who underwent TAA from 2015 to 2020. TAA cases of patients younger than 18 years were excluded. Cases were also excluded if any of the following variables had missing information: age, height, weight, functional status, discharge destination, and preoperative steroid use.

Variables collected in this study included patient demographics, comorbidities, surgical characteristics, and 30-day postoperative complication data. Patient demographics included age, body mass index (BMI), gender, functional health status, American Society of Anesthesiologists (ASA) classification, current smoking status, and preoperative chronic steroid use. Notable preoperative comorbidities included insulin-dependent and non-insulin-dependent diabetes, chronic obstructive pulmonary disease (COPD), congestive heart failure (CHF), hypertension, and bleeding disorders. Surgical characteristics included operative duration. Postoperative complications occurring within 30 days were included in the analysis. These complications included septic shock, sepsis, urinary tract infection, deep vein thrombosis, superficial incisional SSI, organ/space SSI, deep incisional SSI, bleeding transfusions, unplanned intubation, wound dehiscence, myocardial infarction, pulmonary embolism, pneumonia, stroke, ventilator > 48 hours, cardiac arrest, nonhome discharge, readmission, and reoperation.

Patients were divided into chronic steroid and non-steroid groups. The chronic steroid cohort consisted of patients who required regular administration of parenteral or oral corticosteroid medication and received such medication within the 30-day preoperative period. Non-steroid users included patients who received short-term steroids over 10 days or less during the 30-day preoperative period and who received corticosteroids dermally, rectally, or via inhalation.

**Figure 1:**
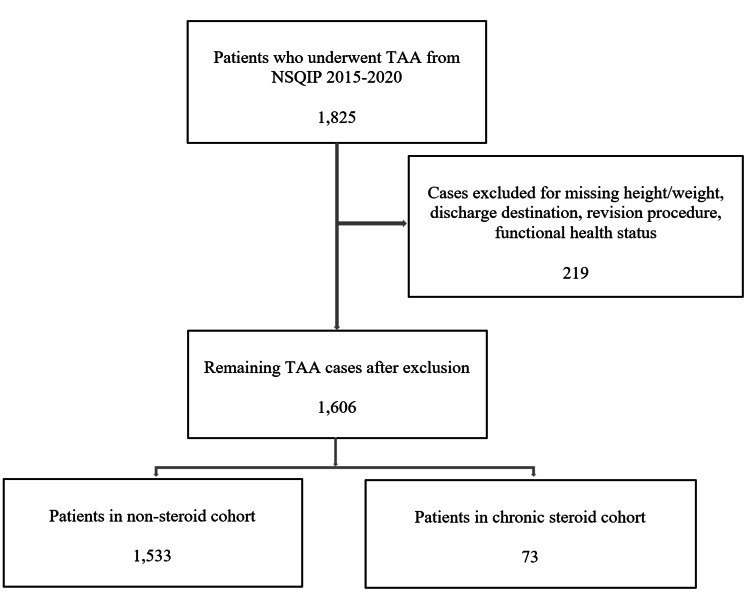
Strengthening the reporting of observational studies in epidemiology (STROBE) diagram with inclusion and exclusion criteria TAA: Total ankle arthroplasty; NSQIP: National Surgical Quality Improvement Program.

A total of 1,825 patients underwent primary TAA in NSQIP from 2015 to 2020. Cases were excluded as follows: eight for missing height/weight, 23 for missing functional health status, one for missing discharge destination, and 187 for revision procedure. Of the 1,606 patients remaining after exclusion criteria, 1,533 (95.5%) patients were included in the non-steroid cohort, and 73 (4.5%) patients were included in the chronic steroid cohort. This is outlined in Figure 1.

All statistical analyses were conducted using the Statistical Package for the Social Sciences (SPSS) software, version 26.0 (IBM Corp., Armonk, NY). Patient demographics and comorbidities were compared between cohorts using bivariate analysis. Multivariate logistic regression, adjusted for all significantly associated patient demographics and comorbidities, was used to identify associations between preoperative chronic steroid use and postoperative complications. Odds ratios (OR) were reported with 95% confidence intervals (CI). The level of statistical significance was set at p < 0.05 [18].

## Results

Bivariate analysis was used to determine patient demographics and comorbidities which were significantly associated with preoperative chronic steroid use (Table 1). The only patient demographics that were significantly associated with preoperative chronic steroid use were female gender (p < 0.001) and ASA ≥ 3 (p < 0.001). The chronic steroid cohort had 67.1% females, compared to 44.0% in the non-steroid cohort group. The chronic steroid cohort also had 63.0% with an ASA classification ≥ 3, compared to 39.6% in the non-steroid cohort group.

**Table 1:** Patient demographics/comorbidities after being separated into non-steroid and chronic steroid use groups Bold p-values indicate statistical significance with p < 0.05 when comparing steroid groups. ASA: American Society of Anesthesiologists; COPD: Chronic obstructive pulmonary disease.

	Non-steroid (n = 1,533)		Chronic steroid (n = 73)		
Characteristics	Number	Percent	Number	Percent	p-value
Total	1533	100.0%	73	100.0%	
Age (years)					0.802
18-39	55	3.6%	3	4.1%	
40-64	715	46.6%	38	52.1%	
65-74	533	34.8%	22	30.1%	
≥75	230	15.0%	10	13.7%	
Body mass index (kg/m^2^)					0.356
<18.5	2	0.1%	0	0.0%	
18.5-29.9	703	45.9%	42	57.5%	
30.0-34.9	451	29.4%	17	23.3%	
34.9-39.9	249	16.2%	11	15.1%	
≥40	128	8.3%	3	4.1%	
Gender					<0.001
Female	674	44.0%	49	67.1%	
Male	859	56.0%	24	32.9%	
Functional health status					0.063
Independent	1523	99.3%	71	97.3%	
Dependent	10	0.7%	2	2.7%	
ASA classification					<0.001
1-2	926	60.4%	27	37.0%	
≥3	607	39.6%	46	63.0%	
Diabetes mellitus					0.283
No	1355	88.4%	63	86.3%	
Non-insulin-dependent	141	9.2%	6	8.2%	
Insulin-dependent	37	2.4%	4	5.5%	
Current smoking status					0.387
No	1405	91.7%	69	94.5%	
Yes	128	8.3%	4	5.5%	
COPD					0.418
No	1494	97.5%	70	95.9%	
Yes	39	2.5%	3	4.1%	
Congestive heart failure					0.999
No	1526	99.5%	73	100.0%	
Yes	7	0.5%	0	0.0%	
Hypertension					0.777
No	719	46.9%	33	45.2%	
Yes	814	53.1%	40	54.8%	
Bleeding disorders					0.418
No	1494	97.5%	70	95.9%	
Yes	39	2.5%	3	4.1%	
Operative duration (min)					0.657
<112	360	23.5%	19	26.0%	
112-180	819	53.4%	35	47.9%	
>180	354	23.1%	19	26.0%	

Bivariate analysis was used to determine postoperative complications associated with preoperative chronic steroid use versus non-steroid use (Table 2). Chronic steroid use was not associated with superficial SSI (p = 0.634) or wound dehiscence (p = 0.999). The only postoperative complication that was significantly associated with preoperative chronic steroid use was sepsis (p = 0.031).

**Table 2:** Bivariate and multivariate analysis of 30-day postoperative complications in patients with and without chronic steroid use Bold p-value indicates statistical significance with p < 0.05 when comparing steroid groups. SSI: Surgical site infection; CI: Confidence interval.

	Non-steroid (n = 1,533)		Chronic steroid (n = 73)		
Postoperative complication	Number	Percent	Number	Percent	p-value
Septic shock	1	0.07%	0	0.00%	0.998
Sepsis	1	0.07%	1	1.37%	0.031
Urinary tract infection	6	0.39%	0	0.00%	0.999
Deep vein thrombosis	6	0.39%	0	0.00%	0.999
Superficial incisional SSI	13	0.85%	1	1.37%	0.643
Bleeding transfusions	1	0.07%	0	0.00%	1.000
Unplanned intubation	3	0.20%	0	0.00%	0.999
Wound dehiscence	6	0.39%	0	0.00%	0.999
Myocardial infarction	1	0.07%	0	0.00%	1.000
Organ/space SSI	6	0.39%	0	0.00%	0.999
Pulmonary embolism	4	0.26%	0	0.00%	0.999
Deep incisional SSI	0	0.00%	0	0.00%	0.999
Pneumonia	2	0.13%	0	0.00%	1.000
Stroke	3	0.20%	0	0.00%	0.999
Ventilator > 48 hours	1	0.07%	0	0.00%	1.000
Cardiac arrest	0	0.00%	0	0.00%	0.999
Nonhome discharge	115	7.50%	4	5.48%	0.521
Readmission	22	1.44%	2	2.74%	0.378
Reoperation	12	0.78%	0	0.00%	0.999
Postoperative complication	Odds ratio	95% CI			p-value
Sepsis	38.46	2.14-690.20			0.013

Multivariate logistic regression analysis was used to adjust the patient variables that were significantly associated with chronic steroid use (female gender and ASA ≥ 3). Multivariate logistic regression identified sepsis (OR: 38.46; 95% CI: 2.14-690.20; p = 0.013) to be independently associated with preoperative chronic steroid use (Table 2).

## Discussion

The most important finding of this study was the lack of association between chronic steroid use and wound dehiscence or superficial SSI. Thirty-day postoperative complications and their association with chronic steroid use in patients who underwent TAA from 2015 to 2020 using a large national database were reported. Our analysis included 1,606 patients, and 73 (4.5%) patients in this group were prescribed steroids for a chronic condition. Through bivariate analysis, we identified chronic steroid use as a possible risk factor for sepsis and found no association between chronic corticosteroid use and superficial SSI or wound dehiscence. After controlling for significantly associated patient demographics and comorbidities, we identified that chronic steroid use does place patients at increased risk of sepsis following TAA.

Due to recent technological advances, there has been a notable resurgence in the use of TAA for the management of end-stage ankle arthritis. Due to a steady increase in the surgical volume of TAA, there is a growing importance of understanding patient risk factors and their effects on patient outcomes. One well-recognized surgical risk factor is chronic corticosteroid use [11,14]. Corticosteroids are potent immunomodulators that exert anti-inflammatory effects through inhibition of key inflammatory pathways (e.g., cyclooxygenase-2, inducible nitric oxide synthase, tumor necrosis factor alpha, and pro-inflammatory cytokines) via direct effects on gene regulation [11,20]. In the setting of autoimmune or inflammatory disease states, these effects prevent inadvertent damage to surrounding structures through a hyper-reactive immune response. However, the effects of corticosteroids also interfere with a wide range of normal metabolic processes, leading to a multitude of common side effects and complications from long-term steroid use [11,20].

Established side effects of chronic steroid use include impaired wound healing, increased susceptibility to infection, osteopenia/osteoporosis, impaired glycemic control, avascular necrosis, and muscle atrophy [11,14,20]. These side effects may significantly impact postoperative outcomes. Thus, patients with chronic autoimmune or inflammatory diseases that require chronic corticosteroid use need close monitoring and careful evaluation before surgery [11]. Within the orthopedic community, current literature has established associations between chronic steroid use and adverse outcomes following total hip, knee, and shoulder arthroplasties [12,18]. However, there is a paucity of literature surrounding chronic steroid use and TAA. Our study addresses this gap in the literature through the use of a large national database.

Our study found that chronic steroid use in patients who underwent TAA was associated with female gender and an ASA classification ≥ 3. These discrepancies in patient gender and ASA classification are not particularly surprising. First, steroid use is a cornerstone in the management of autoimmune diseases, which has a higher prevalence in females compared to males [21]. Second, chronic steroid use is typically reserved for patients with severe inflammatory disease that is refractory to other therapeutic modalities [20]. This level of disease severity would meet the criteria for ASA classification ≥ 3, which is described as a “patient with severe systemic disease” [22]. Interestingly, after controlling these factors and based on the available data, chronic steroid use was not identified as an independent risk factor for postoperative superficial SSI or wound dehiscence after TAA.

Our finding that preoperative chronic steroid use does not increase the risk of superficial SSI or wound dehiscence following TAA is not completely consistent with previous studies in total joint arthroplasty. This is possibly due to the differing causes for end-stage ankle arthritis compared to other joints treated with arthroplasty. A study by Poss et al. reported that patients with rheumatoid arthritis treated with chronic steroids who underwent total joint arthroplasty had an increased risk of postoperative infection and sepsis that extended years after their procedure [23]. Another study on total hip arthroplasty by Boddapati et al. demonstrated that patients with chronic steroid use had roughly twice the 30-day readmission rate compared to patients without chronic steroid use. Importantly, infections accounted for approximately one-third of these readmissions [10]. A major cause for our findings may be a cause for end-stage ankle arthritis being largely caused by previous trauma. Notably, there was a relatively small proportion of patients using steroids chronically, which may reflect an overall healthier cohort.

In a similar study looking at chronic steroid use in total shoulder arthroplasty, Ling et al. reported chronic steroid use as a significant risk factor for septic shock within 30 days postoperatively [18]. This finding is consistent with one of our findings that chronic steroids may lead to an increased risk of sepsis. However, with only one case of sepsis seen in the chronic steroid cohort, this correlation may need further investigation. Notably, Ling et al. also did not find a significant correlation between chronic steroid use and superficial SSI or wound dehiscence [18]. Additionally, our findings are consistent with a recent meta-analysis and systematic review conducted by Mousavian et al., which demonstrated comparable complication rates between inflammatory and noninflammatory arthritis patients undergoing TAA [24]. This meta-analysis supports our conclusion that TAA is a safe and effective procedure in patients who may be taking chronic steroids for inflammatory arthritis.

This study was constrained by some inherent limitations of using the NSQIP database. One major limitation of the study is the limited number of cases involving patients with chronic steroid use. Further investigation is needed to determine if sepsis is associated with chronic steroid use over a larger population of users. In addition, although the use of TAA has been increasing, it is still relatively uncommon with respect to other joint arthroplasties, resulting in a relatively smaller sample size. Additionally, the NSQIP database does not provide information on the indication for chronic steroid use. Therefore, we were unable to account for the disease for which chronic steroids were prescribed. Furthermore, the postoperative complications recorded in NSQIP are only limited to the 30-day postoperative period. As indicated in the study by Poss et al., infectious complications following TAA may extend to years beyond the procedure, so a 30-day postoperative window may not be representative of the adverse effects of chronic steroid use [23]. Additionally, the study is limited by a possibly different presentation of SSI due to chronic steroid use. Due to the suppression of their immune system in combination with the 30-day window, SSI may be missed in some of these patients. This may also result in an increased risk of sepsis that is not observed.

Although this study is not without limitations, a large national database was utilized to identify patients undergoing TAA and analyze postoperative complications associated with preoperative chronic steroid use. While patients may need further investigation for risk of sepsis, chronic steroid use should not be seen as a contraindication for the procedure. As the surgical volume for TAA continues to grow, further studies are needed to better assess both sepsis risk with a larger cohort and long-term postoperative complications associated with chronic steroid use.

## Conclusions

Preoperative chronic steroid use is not a risk factor for superficial SSI or wound dehiscence within 30 days following TAA. Chronic steroid users who underwent TAA were more likely to be female with an ASA ≥ 3. As TAA continues to become a more attractive alternative to AA, a better understanding of preoperative risk factors can aid in widening indications and knowing who are truly at risk for complications.
